# Draft Genome Sequence of a Multidrug- and Colistin-Resistant *mcr-1*-Producing Escherichia coli Isolate from a Swine Farm in Mexico

**DOI:** 10.1128/genomeA.00102-18

**Published:** 2018-03-08

**Authors:** Ulises Garza-Ramos, Elsa Tamayo-Legorreta, Doris María Arellano-Quintanilla, Nadia Rodriguez-Medina, Jesús Silva-Sanchez, Juan Catalan-Najera, Marisol Karina Rocha-Martínez, María Asunción Bravo-Díaz, Celia Alpuche-Aranda

**Affiliations:** a Instituto Nacional de Salud Pública (INSP), Centro de Investigación Sobre Enfermedades Infecciosas (CISEI), Cuernavaca, Mexico; b Centro Nacional de Servicios de Constatación en Salud Animal del Servicio Nacional de Sanidad, Inocuidad y Calidad Agroalimentaria, Mexico City, Mexico

## Abstract

A colistin-resistant *mcr-1*-carrying Escherichia coli strain, RC2-007, was isolated from a swine farm in Mexico. This extraintestinal and uropathogenic strain of E. coli belongs to serotype O89:H9 and sequence type 744. Assembly and annotation resulted in a 4.9-Mb draft genome that revealed the presence of plasmid-mediated *mcr-1*-IS*ApI1* genes as part of a prophage.

## GENOME ANNOUNCEMENT

The plasmid-borne *mcr-1* gene that confers colistin resistance was first described in China in both animals and humans and currently is of great concern to public health (1). PCR screening of Escherichia coli isolates from a collection of swine stool samples from a farm in Mexico in 2015 (2) revealed that E. coli strain RC2-007, which was obtained from a 2-month-old healthy male piglet and which produces extended-spectrum β-lactamase, acquired the *mcr-1* gene.

A total genomic sample of E. coli strain RC2-007 was extracted and purified using the DNeasy kit (Qiagen). The whole-genome sequence was generated using pyrosequencing on the 454 Roche FLX Titanium platform. The reads were assembled into 167 contigs with 20-fold coverage using Newbler software version 2.7 (Roche). The draft bacterial genome sequence comprised an estimated 4,934,540 bp, and gene prediction and annotation were carried out using the bioinformatic MicroScope platform (3). A total of 4,788 coding DNA sequences and 64 tRNAs were identified.

The whole-genome sequence was subjected to *in silico* analysis (4–7). The following families of antibiotic resistance genes were identified: *mcr-1* (polymyxin E); CTX-M-55 and TEM-1 (β-lactams); *aac(3)-IIa*, *strA-B*, *aadA5*, and *aadA12* (aminoglycosides); *mph* (macrolide); *lnu* (lincosamide); *catA1* and *florR* (phenicol); *sul1-2-3* (sulfonamide); *tet-B* (tetracycline); and *dfrA17* (trimethoprim). The GyrA and ParC proteins were analyzed, which led to the identification of the mutations Ser83Leu and Asp87Asn for GyrA and the mutations Thr56Ala and Ser80Iso for ParC.

The virulence factor genes identified were *gad* (glutamate decarboxylase), *cma* (colicin M), extraintestinal and uropathogenic *iucC* (siderophore), and *fimH* (type 1 fimbriae), but no enteric pathotype genes were detected. The E. coli RC2-007 chromosome carried genes for serotype O89:H9 and sequence type 744, which interestingly was previously described in E. coli isolates obtained from gulls in Ushuaia, Argentina, that carried both the *mcr-1* gene and CTX-M alleles (8).

The plasmid profile of the E. coli RC2-007 isolate is of 120- and 100-kb plasmids. The *bla*_CTX-M-55_ and *mcr-1* genes are encoded, respectively, on conjugative 120-kb and nonconjugative 100-kb plasmids, this situation could have resulted from two independent events in the acquisition of resistance to both colistin and cephalosporin. Although identification of the incompatibility group of the pRC2-007 (100-kb) plasmid was unsuccessful by PCR-based replicon typing (9), the presence of Incp0111 (*repA* gene), with 98.64% nucleotide identity, was identified *in silico* (10).

Genetic analysis of the *mcr-1* gene was performed by PCR and *in silico*. The insertion sequence IS*Apl1*, upstream of the *mcr-1* gene, was identified by PCR (ISApl1-F 5′-TGATGAGTACTTCCTACCGACA-3′ and CLR5-R [1]). Analysis of the genome with PHAST (11) confirmed the presence of the IS*Apl1-mcr-1* genes in the 49,379-bp prophage, which is part of the 100-kb pRC2-007 plasmid. Additional work is required to determine the implication of the prophage in *mcr-1* gene dissemination.

The first identification of an *mcr-1* gene in a multidrug-resistant E. coli strain isolated from swine stool samples in Mexico opens the possibility of potential dissemination in human and veterinary medicine with future clinical implications.

### Accession number(s).

The annotated genome sequences reported here are available at the European Nucleotide Archive under the accession numbers FUEI01000001 to FUEI01000167.
